# 27 Pain Medication Use at Follow up Is Associated with Long-term Outcomes

**DOI:** 10.1093/jbcr/irac012.030

**Published:** 2022-03-23

**Authors:** Brian M Kelter, Lauren J Shepler, Barclay T Stewart, Steven E Wolf, Samuel P Mandell, Lewis E Kazis, Colleen M Ryan, Jeffrey C Schneider

**Affiliations:** Spaulding Rehabilitation Hospital, Boston, Massachusetts; Spaulding Rehabilitation Hospital, Boston, Massachusetts; University of Washington, Seattle, Washington; University of Texas Medical Branch at Galveston, Galveston, Texas; UT Southwestern, Parkland Regional Burn Center, Dallas, Texas; Boston University School of Public Health, Spaulding Rehabilitation Hospital, Harvard Medical School, Boston, Massachusetts; Harvard Medical School, Boston, Massachusetts; , Massachusetts

## Abstract

**Introduction:**

Use of prescription pain medication after burn injury is commonly required. However, little is known about long-term pain medication use and its association with outcomes. Therefore, the purpose of this study is to assess patterns of prescription pain medication use after discharge and the association between these medications and quality of life outcomes.

**Methods:**

Data from the Burn Model System National Longitudinal Database (2015-2021) were analyzed. Pain medication use was assessed at pre-injury (recall at discharge), discharge (medical record) and follow-up (self-report at 6, 12, and 24 months after injury). Outcome measures included: VR-12 Physical and Mental Component Summary scores (PCS and MCS), Community Integration Questionnaire (CIQ), Posttraumatic Stress Disorder Checklist (PCL), Satisfaction with Life Scale (SWLS), and NeuroQOL Stigma. The population was divided into two groups, those taking and not taking prescription pain medications at one year. Regression analyses examined associations between prescription pain medication use and outcomes at 12 months, controlling for age, gender, race, ethnicity and burn size.

**Results:**

Of the 645 participants, 15% reported prescription pain medication use prior to their burn. At discharge, 81% reported use of an opioid and 46% reported use of a neuropathic pain medication. At 12 months, 32% of individuals indicated prescription pain medication use. The pain medication group exhibited larger burn size (24.0% vs 15.2%) and longer hospital stays (40.4 vs 25.0 days) than the non-pain medication group (p< 0.0001 for all). Additionally, 25% of individuals who reported pre-injury pain medication use also reported use at 12 months. Regression analyses demonstrated that pain medication use was associated with worse physical health (PCS: coefficient 8.69, p< 0.0001) mental health (MCS: 6.31, p< 0.0001), stigma (NeuroQOL Stigma: 3.91, p< 0.0001), and satisfaction with life (SWLS: -3.66, p< 0.0001) at one year. Additionally, pain medication use was associated with 45% decreased odds of being employed (coefficient 0.55, p=0.029) and approximately 3 times greater odds of having post-traumatic stress disorder at 12 months (coefficient 3.25, p< 0.0001).

**Conclusions:**

There are significant associations between prescription pain medication use and worse physical, mental and employment outcomes at twelve months. This information may be used to trigger screening and manage long-term recovery outcomes.

